# Ultrasonic Transducer for Non-Destructive Testing of Structures Immersed in Liquid Sodium at 200 °C †

**DOI:** 10.3390/s19194156

**Published:** 2019-09-25

**Authors:** Jean-François Saillant, Régis Marlier, Frédéric Navacchia, François Baqué

**Affiliations:** 1 INTERCONTROLE/Framatome, 4 Rue Thomas Dumorey, 71100 Chalon-sur-Saône, France; 2 Framatome, 10 rue Juliette Récamier, 69456 Lyon, France; regis.marlier@framatome.com; 3 C.E.A. (Commissariat à l’Energie Atomique et aux Energies Alternatives), Centre de Cadarache, DEN/DTN/STCP/LISM, 13108 St Paul Lez Durance, France; frederic.navacchia@cea.fr (F.N.); francois.baque@cea.fr (F.B.)

**Keywords:** ultrasonic transducer, NDT, NDE, sodium, reactor, ultrasound, TUCSS, ISI&R, SFR, FBR

## Abstract

TUCSS transducer (French acronym standing for Transducteur Ultrasonore pour CND Sous Sodium) is designed for performing NDT (Non-Destructive Testing) under liquid sodium. Under sodium, the tests results obtained show that these transducers have sufficiently good acoustic properties to perform basic NDT of a structure immersed under liquid sodium at about 200 °C using conventional immersion ultrasonic technics. Artificial defects were made next to an X-shaped weld and could clearly be detected.

## 1. Introduction

Today, nuclear power provides about 10% of the world’s electricity [1], principally generated from thermal–neutron reactors such as Pressurized Water Reactors (PWR) or Boiling Water Reactors (BWR). Parallel to the development of these thermal reactors, several countries have undertaken programs on Sodium Fast Reactor (SFR) development. To date, 12 experimental prototypes and six commercial-size reactors of SFRs have been designed, constructed, and operated [2]. SFRs offer great potential for a sustainable nuclear energy for many reasons, such as increased uranium utilization, fast nuclear power growth from the available resources through the breeding of fissile material, reduction of radioactive waste, utilization of fission products and other radioactive isotopes, higher thermodynamic efficiency.

Regardless of these advantages, SFRs pose specific problems related to the use of chemically reactive sodium. For instance, looking at the available feedback experience of SFRs operation, In-Service Inspection and Repair (ISI and R) is a significant issue to deal with and plays an important role in the safety approach of the plant for complying with new safety standards [3]. Non-destructive Testing (NDT) of the components immersed in liquid sodium is particularly challenging because of the harshness of the environment: high temperature, chemical aggression, and irradiation. The opacity of the medium is also a specificity, which makes ultrasonic testing a well-adapted candidate for performing NDT inside the primary circuit of these reactors.

This article reports on the feasibility of an ultrasonic transducer designed for performing NDT on a welded structure immersed in liquid sodium at approximately 200 °C. The design and fabrication of the ultrasonic transducer is first described, as well as the whole methodology for carrying out this specific ultrasonic inspection experiment. Finally, experimental results show that the developed transducer has sufficient acoustic properties to be considered for performing NDT during the outages of an SFR.

## 2. Ultrasonic Transducer for Non-Destructive Testing (NDT) under Liquid Sodium

Ultrasonic transducers for NDT under liquid sodium have to respond to very particular specifications. Technical difficulties to be overcome are first described before presenting the design of the Transducteur Ultrasonore pour CND Sous Sodium (TUCSS) transducer.

### 2.1. Technical Difficulties to be Overcome

#### 2.1.1. Temperature Resistance

ISI and R of the reactor will be performed during outages, as sodium temperature is maintained at about 200 °C. This temperature is not conventional for ultrasonic transducers, and, moreover, the transducer has to withstand this temperature from several hours to several days, continuously. The transducer has to be functional at this temperature without external cooling, as it can be immersed under 10 m, or more, of sodium, which makes cooling of the transducer inefficient with such a depth.

Several technologies can be considered for generating ultrasound. The choice here was made to use piezoelectric transduction for converting electrical energy into ultrasonic pressure waves, and vice versa. If the piezoelectric crystal used is ferroelectric, it is important to pay attention to its Curie temperature so that it preserves its piezoelectric properties at the temperature of interest.

Passive materials conventionally used in NDT transducers fabrication cannot be used at 200 °C, as their physical integrity could be damaged. Moreover, thermal expansion coefficients of the materials of the different components (backing, matching layers, piezoelectric material, etc.) lead to different strains at interfaces, generating stresses which may cause bond failure and therefore malfunction of the transducer.

Electrical connections are also affected by the temperature level. So, cabling and soldering have to be chosen carefully.

#### 2.1.2. Chemical Compatibility

It was mentioned in the introduction that sodium was chemically reactive (like other members of the alkaline elements family, it is a powerful reducing agent). It is reactive to air and water but it can also be aggressive to different materials, so much care has to be taken at the external conditioning of the probe. For instance, epoxy resins are not all compatible with sodium. Obviously, all materials used for the external packaging of the probe must be compatible with sodium.

#### 2.1.3. Irradiation Resistance

Transducers will be exposed to high levels of gamma irradiation (there is no neutron irradiation, as inspections occur during reactor outage). Indeed, the potentially inspected structures can be quite close to the reactor core, and thus activated by the neutron flux during power generation cycles. Our objective is to design a transducer capable of withstanding a cumulated dose up to 216 kGy, corresponding approximately to a one-month inspection campaign underneath the SFR core.

#### 2.1.4. Acoustic Performances Compatible with Non-Destructive Testing (NDT) Requirements

Short ultrasonic pulses are necessary in order to have sufficient resolution for efficient NDT inspection. Controlling the pulse shape is necessary and our objective is to have a ringdown of a maximum of 4 cycles at −20 dB at operating resonant frequency. Also, the dynamics of the transducer have to be fairly high in order to be able to sense echoes from potential flaws inside the matter. In a first approach, we aim to have signal to noise ratio (SNR) > 50 dB on the entrance echo of a flat stainless-steel block.

The acoustic properties of sodium are relatively similar to that of water, even considering that sodium is a metal in its pure state. At 200 °C, its properties are [4]:

- Density = 903.5 kg/m^3^
- Sound velocity = 2471.8 m/s
- Acoustic impedance = 2.23 MRayl

As a consequence, the same design rules can be applied as those for underwater immersion NDT transducer design. The problem mainly consists in finding suitable backing and matching layer materials for operating at a continuous temperature of 200 °C.

The most critical acoustic issue to be addressed is the acoustic transmission at the interface between sodium and the transducer’s emissive face. Indeed, liquid sodium does not wet all material surfaces. By comparison, it has a similar behavior to a drop of mercury which remains more or less spherical when put in contact with a regular table, rather than spreading flat like a drop of water would do. When a liquid does not wet a surface, it means that there remains a thin film of gas in between. Transmission of acoustic energy between solid/gas or between liquid/gas is very inefficient in the MHz range; the acoustic transmission coefficient is in the order of 0.001%, compared to optimal state. This means the transducer’s emissive face should be made from a material that can be wetted by sodium at 200 °C. Again, temperature can be a parameter of influence in the wetting of materials. For example, sodium does not wet stainless steel at 200 °C; however, it wets well at a temperature above 400 °C, as surface oxides react with sodium [5]. Note that when stainless steel wetting by sodium is achieved, it remains wetted even if temperature decreases.

### 2.2. Transducer Design and Fabrication

Several transducer technologies have been developed for under sodium viewing. Most of them are listed in the review paper [6,7,8,9,10,11,12]. EMAT (Electromagnetic acoustic transducer) technology also seems to be interesting, due to the fact that wetting is not necessary to generate or receive ultrasonic waves [13]. Nevertheless, NDT is very demanding in terms of acoustic performances because of the need to satisfy to both constraints of short pulse lengths and high signal dynamics. To date, and to the authors’ knowledge, no transducer technology has proven capable of performing conventional NDT in immersion under liquid sodium at 200 °C.

**Figure 1:**
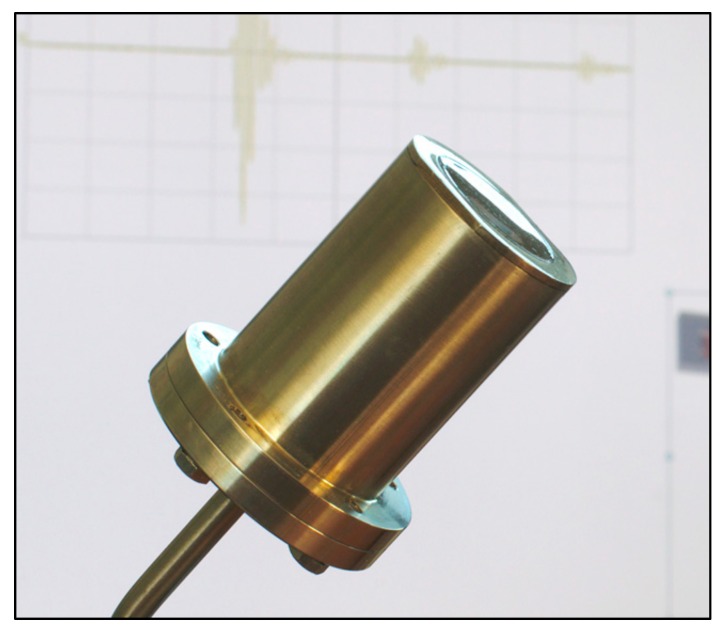
Photograph of a “Transducteur Ultrasonore pour CND Sous Sodium” (TUCSS) transducer.

TUCSS transducer (French acronym standing for Transducteur Ultrasonore pour CND Sous Sodium) has been designed and fabricated at INTERCONTROLE/Framatome. Its main objective is to demonstrate the feasibility of performing conventional NDT under the technical constraints listed in Section 2.1. A photograph of a TUCSS transducer is shown in Figure 1.

**Figure 2:**
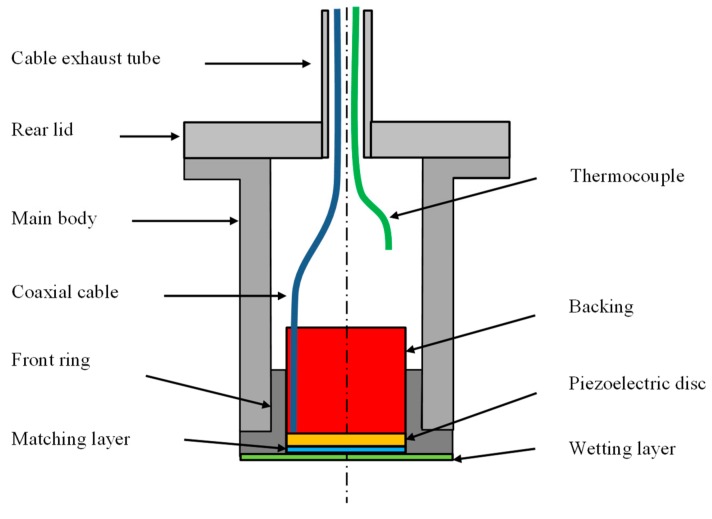
Schematic representation of the structure of a Transducteur Ultrasonore pour CND Sous Sodium (TUCSS) transducer.

TUCSS is based on piezoelectric transduction technology for generating and sensing ultrasonic waves. A schematic representation of the transducer is shown in Figure 2. Its characteristics are as follows:

The piezoelectric disc is made from a PZT (Lead Zirconate Titanate) ceramic NAVY type II that has a Curie point of 350 °C. Its free resonant frequency is 2.2 MHz and has an active diameter of 20 mm.

The backing material is made from a mixture of attenuative high temperature elastomers and large size particles. Its roles are to damp the vibration of the piezoelectric disc and to attenuate the acoustic energy travelling backwards (so that there are no spurious echoes originating from inside the transducer). Its thickness is approximately 20 mm.

The matching layer is made from low attenuation high temperature thermosetting resin. Its role is to optimize the transfer of acoustic energy travelling forward. Its thickness is 250 µm and its acoustic impedance at 200 °C is 2.53 MRayl.

The front layer used for wetting in sodium is made of elastomer. Its thickness is 70 µm and its acoustic impedance at 200 °C is 0.59 MRayl. Its properties were measured using the technique in Reference [14].

The cable is custom PFA (Perfluoroalkoxy) coaxial cable.

A type K thermocouple is integrated to monitor the temperature inside the transducer during testing and during pre-heating of the transducer before immersion (to avoid thermal shocking).

All the mechanical parts (main body, front ring, and rear lid) are made from 316 L stainless steel. The length from the inside face of the shoulder to front face of the transducer is 50 mm. The diameter of the body is 36 mm.

## 3. Materials and Methods

Prior work using TUCSS transducers [15] reported on experiments related to the “contact” NDT technique. The transducer was brought into contact with the testing block, as the whole setup was immersed in liquid sodium. The initial result was positive, as a ∅ 3 mm side drilled hole (SDH) could be detected in the matter. However, it was found that the friction between the two contact surfaces was causing the loss of wetting on both surfaces (transducer and inspected block).

Further work using TUCSS transducers reported on “immersion NDT technique” [16], where the transducer was shifted away from the surface of the inspected part (i.e., not in contact). The results were satisfactory and it was decided to pursue this direction for the NDT of welded assemblies of SFR structures. Immersion NDT techniques are very common in the industry [17], and especially in the nuclear industry [18].

The present NDT experiment was conducted on a 316L stainless steel block. Here we considered a structure representing two 40 mm thick plates welded together by X-shape Manual Shielded Metal Arc Welding (Manual SMAW).

**Figure 3:**
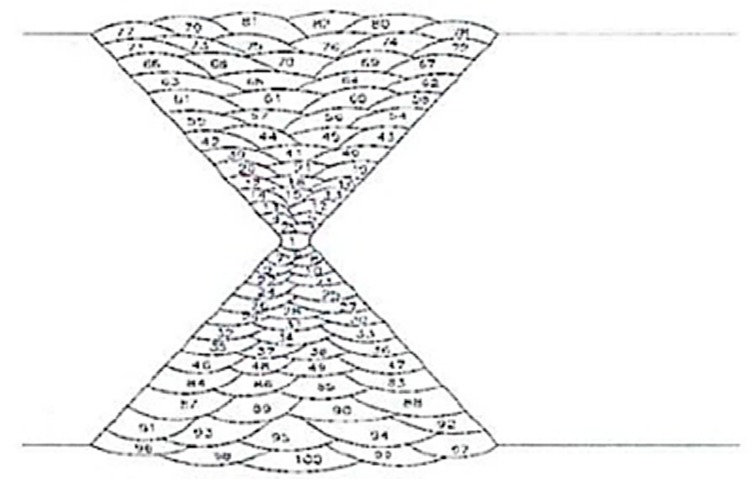
Multiple passes of the X-shape Shielded Metal Arc Welding (SMAW) weld.

Austenitic welds add a great complexity to the NDT inspection of a structure. The different passes (see Figure 3) and the resulting recrystallized microstructures cause local heterogeneities in the stiffness matrix, which disturb and scatter the ultrasonic wave. The aim of the present study is to determine if it is possible to detect a flaw placed beyond the weld, in immersion under liquid sodium at 200 °C.

**Figure 4:**
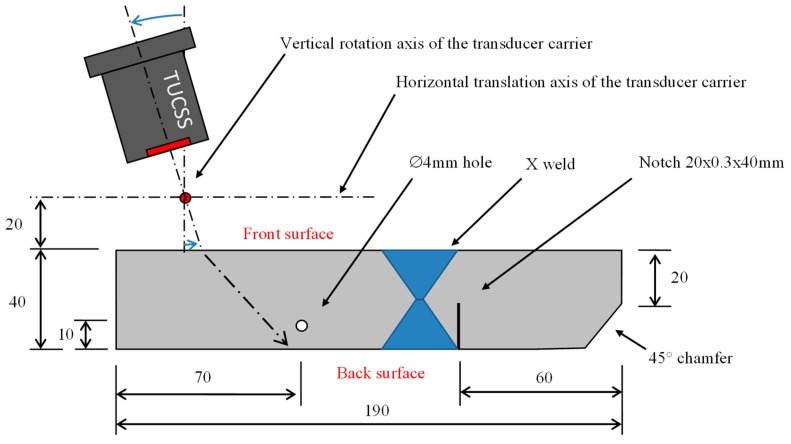
Schematic representation of the test block (horizontal cross section).

The test block considered in this experiment is represented in Figure 4. It consisted of a 190 × 40 mm block of 316 L stainless steel, with a height of 100 mm. It can be seen that it included the X-shape weld and a 20 × 0.3 mm notch (with a notch height of 40 mm). This notch is made by spark machining from the back surface and is placed at mid-height of the block. Two other indications were added to the block in order to have reference points: a ∅ 4 mm side hole (drilled all the way through the height of the block) and a 45° chamfer.

**Figure 5:**
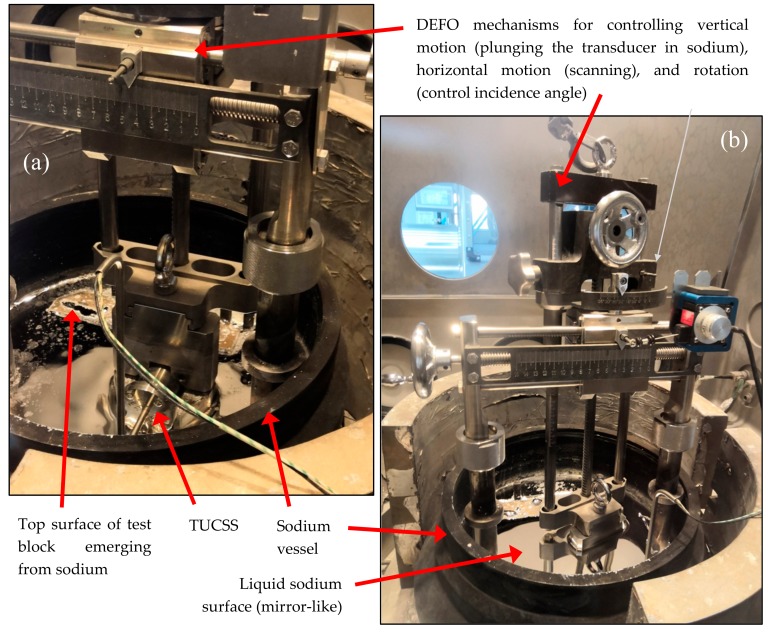
Photographs of experiment (**a**) before immersion of the transducer and (**b**) when the transducer is immersed.

Under-sodium tests were conducted in a thermally regulated cylindrical vessel (∅ 320 × 200 mm) containing 10 L of liquid sodium (pure sodium fusion temperature is 98 °C). A characterization device, called DEFO (Dispositif d’Essais Faisceau Oblique), was specifically designed and fabricated in order to accurately move the TUCSS in front of the test block inside this sodium filled vessel. Photographs of DEFO placed in the sodium vessel can be seen in Figure 5.

Its mechanisms are made for translating and rotating the transducer and are kept as simple as possible. They allow the transducer to move according to the 3 following motions:

Vertical translation for plunging of the transducer into liquid sodium.

Horizontal motion along the translation axis (see Figure 4) for performing the ultrasonic scan.

Rotation around the vertical axis (see Figure 4) to control the incidence angle with respect to the front surface of the test block. This rotation applies to the transducer carrier and its axis is placed 20 mm away from the front surface. The carrier can keep its orientation when it is moved along the horizontal translation axis. The transducer’s front face is placed 25 mm away from the rotation axis.

All the motions are manually controlled. Motors were excluded because of increased complexity brought by inert argon atmosphere (electrical arcing), presence of sodium aerosols, high temperatures, and instrumentation of the existing glove box. This experiment was conducted in a glove box of CEA-DEN (Cadarache, France) sodium facilities.

Acquisitions are made with a TabletUT ultrasonic electronic system (Mistras Eurosonic, Vitrolles, France). The electronic system was connected to a POSIWIRE WS42-1000-6-IE24LI-2-LF (ASM, Moosinning, Germany) wire coder for correlating ultrasonic data with positions along the horizontal scan.

Preparation of the experiment began by cleaning all the parts of the test device with alcohol, followed by complete drying at 120 °C for 24 h. The device was then entered into the glove box and placed into the sodium vessel. The volume of sodium was adjusted to reach the top surface of the test block. At this stage, the TUCSS transducer was not mounted on the DEFO device. The temperature of sodium then rose to 400 °C for 6 h. This temperature step is compulsory for the sodium to wet the stainless-steel test block and, consequently, for ultrasonic waves to be able to penetrate it (and to be representative of reactor conditions where all internal structures have been wetted by sodium during the initial high temperature cycle). Once the sodium cooled down to 200 °C, the TUCSS was mounted on the transducer carrier, still above the sodium surface, which was then translated down until it touched the surface of sodium (Figure 5a). This allows for a slow pre-heating of the transducer, thus avoiding thermal shocking. The transducer was plunged under the free surface of liquid sodium (Figure 5b) once its thermocouple read a stable temperature (approximately 170 °C). Echoes from the block were visible straight away, indicating that wetting was immediate. The system was then ready for performing ultrasonic scans.

## 4. Results

Several scans have been performed for different incidence angles. Each incidence angle allows the indications inside the test block to be seen with a different detection mode. We first present the normal incidence condition, which makes it easier to familiarize oneself with the reading of the data, and then we present the two incidence angle conditions for which best detection results are obtained.

### 4.1. Normal Incidence Condition

**Figure 6:**
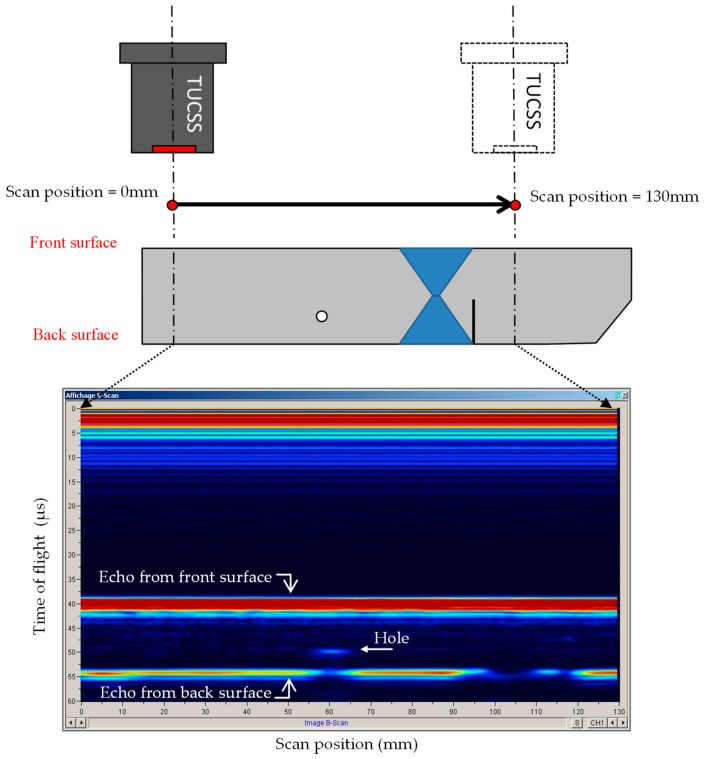
Normal incidence scan.

Figure 6 shows a scan done in normal incidence (i.e., TUCSS axis perpendicular to the surface of the test block). 

This scan can be interpreted as follows:

The trace from 0 to 12 µs is the saturated dead zone of the transducer.

The linear trace at 40 µs is the echo coming from the front surface of the block. It is present at all scan positions from 0 mm to 130 mm.

The spot trace at [60 mm, 50 µs] is the echo coming back from the ∅ 4 mm hole.

The linear traces at 55 µs are echoes coming from the back surface of the block. This backwall echo is not visible at all scan positions:

- It is shadowed by the hole at position 60 mm,
- Between 95 mm and 110 mm, the ultrasonic beam is disrupted/attenuated by the weld, preventing echoes from coming back,
- Between 115–120 mm, the local disruption in the backwall echo is caused by the presence of the notch.

The settings for this scan were as follows:

- Sodium temperature = 200 °C.
- 2 MHz–100 V single square wave excitation.
- 30 dB gain in reception, in order to have the backwall echo without saturation (it was 12 dB for having the front face echo not saturated).

This scan shows that the weld has an important impact on the propagation of ultrasound, as the detection of the backwall echo is really disturbed. Also, even though a 0° incidence should not be used for detecting a notch made perpendicular to the back surface, this scan shows that the shadowing of the backwall echo gives us an indication of its presence.

### 4.2. Oblique Incidence Condition—Use of Shear 45° Waves

**Figure 7:**
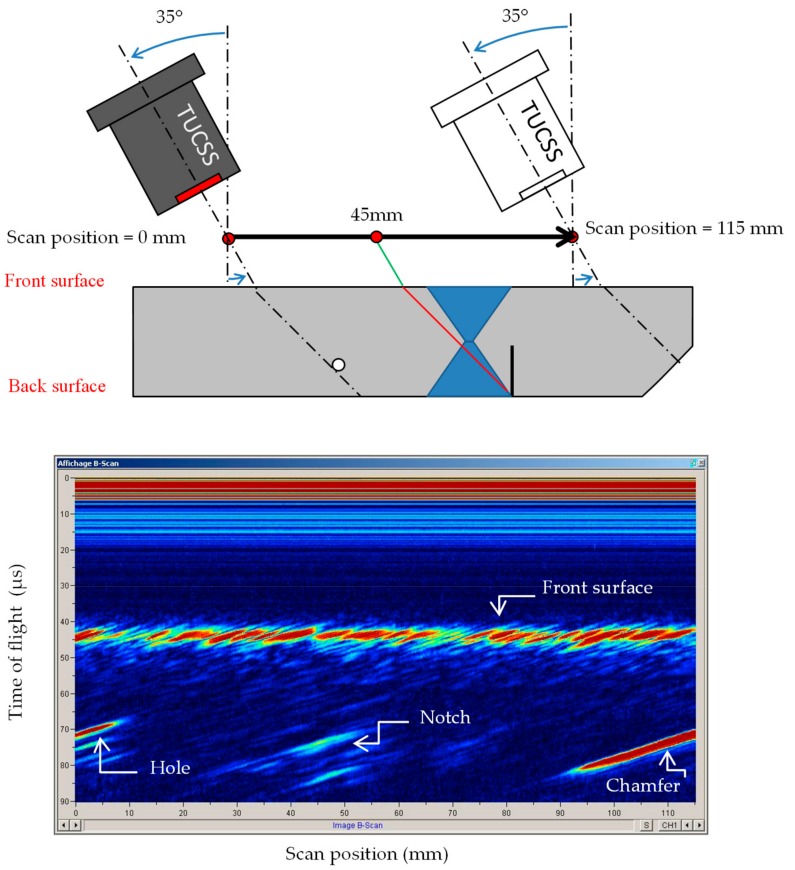
Scan at an incidence of 35°, generating 45° shear waves in the block.

The first critical angle is reached at an incidence angle of 25.53°. Beyond this angle, the entire incident wave in sodium is converted into shear wave in the test block. The notch can be detected using pure shear wave mode. It was found that the best amplitude is obtained at an incidence angle of 35°, producing 45° shear waves (SW45°) in the test block. Figure 7 shows the resulting scan at 35° incidence angle.

The settings for this scan were as follows:

- Sodium temperature = 200 °C.
- 2 MHz–100V single square wave excitation.
- 45 dB gain in reception.

This scan can be interpreted as follows:

The traces from 0 to 20 µs are the saturated dead zone of the transducer. It is longer than the dead zone seen for the scan at normal incidence because the reception gain is 15 dB higher.

The traces at 45 µs all across the scan are echoes coming from the front surface of the block. 

The trace at [5 mm, 70 µs] is the echo coming back from the ∅ 4 mm hole.

The 45° chamfer is represented by the linear trace going from [95 mm, 80 µs] to [115 mm, 70 µs].

Traces at [45 mm, 75 µs] to [48 mm, 85 µs] are echoes originating from the notch.

### 4.3. Oblique Incidence Condition—Use of LLS Detection Mode

**Figure 8:**
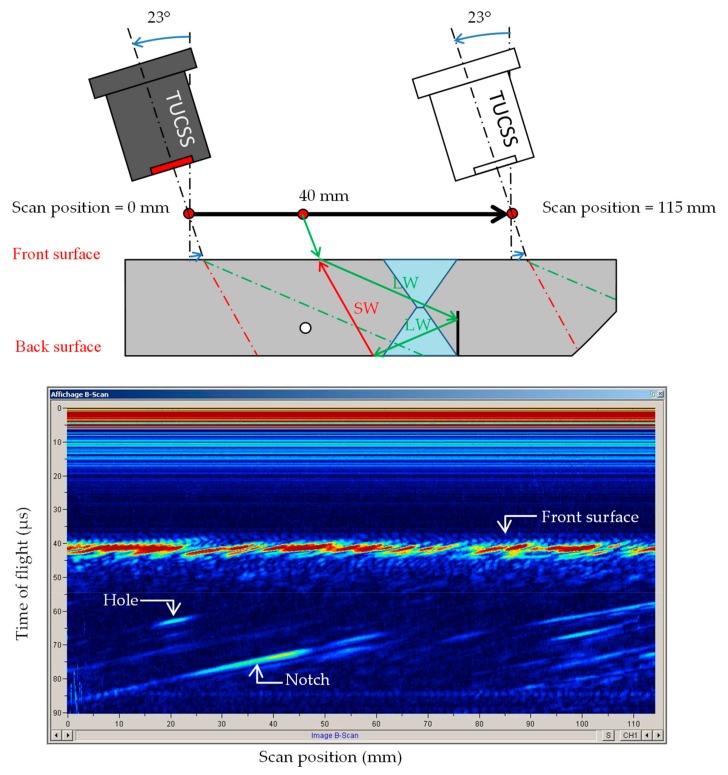
Scan at an incidence of 23°, generating LW65° and SW29° in the block.

When the incidence angle is lower than the first critical angle (25.53° in the present case), the incident wave is converted into two waves with different polarizations: a longitudinal wave (LW) and a shear wave (SW). These two waves have different propagation velocities and their refraction angles are therefore different (see Section 5 for wave velocities measurements). In this study, it was found that an incidence angle of 23° gave the best result for detecting the notch. At this angle, the two waves generated are LW65° and SW29°. Figure 8 shows the resulting scan at 23° incidence angle.

The settings for this scan were as follows:

- Sodium temperature = 200 °C.
- 2 MHz–100 V single square wave excitation.
- 45 dB gain in reception.

This scan can be interpreted as follows:

The traces from 0 to 20 µs are the saturated dead zone of the transducer. It is again longer than the dead zone seen for the scan at normal incidence because the reception gain is again 15 dB higher.

The traces at 40 µs all across the scan are echoes coming from the front surface of the block. 

The trace at [20 mm, 65 µs] is the echo coming back from the ∅ 4 mm hole, detected in direct SW29°.

The notch is represented by the linear trace going from [25 mm, 80 µs] to [45 mm, 75 µs]. It is detected in LLS reflections mode. The first refracted LW65° is reflected off the notch in LW65°, which is then reflected off the backwall in SW29°. The resulting wave comes out in in sodium at the same position and angle (LW23°) as the incident wave.

The 45° chamfer cannot clearly be detected at this incidence angle. It produces some noise visible in 90 µs–115 µs range.

## 5. Discussion

### 5.1. Acoustic Performances

The results shown in Section 4 clearly indicate that it is possible to detect a notch placed beyond the weld using a 2 MHz TUCSS transducer. Immersion testing was performed at different incidence angles, which allowed the notch to be detected with different modes. The best results were obtained using direct SW45° mode and LLS mode using an initial LW65°. In both modes, echoes from the notch were detected with a 45-dB gain for a 100 V excitation at 2 MHz. This is 33 dB higher than the gain required to detect the front surface echo without saturation.

**Figure 9:**
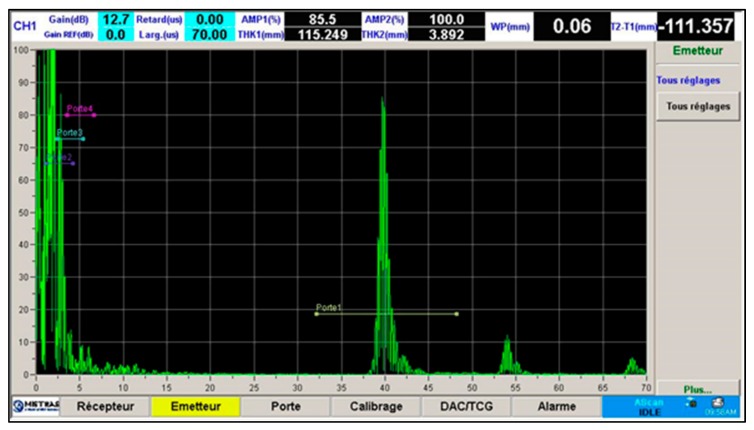
A-scan at incidence = 0°, scanning position = 30 mm, sodium temperature = 200 °C.

The resolution of the transducer was also found to be sufficient. Echoes typically had an approximately 2-µs length, corresponding to a pulse ringdown of approximately 4 wavelengths at 2 MHz. Figure 9 shows a typical A-scan made with 0° incidence angle. The main echo at 40 µs is the echo from the front surface of the test block and the weaker echoes are echoes from the backwall.

These acoustic properties distinguish themselves from those of the other transducer by the resulting combination of high sensitivity and high damping. We attribute this to the fact that most of the transducers fabricated up to date for under sodium operation include a metallic front face. Stainless steel has an acoustic impedance of approx. 47 MRayl, while sodium has an acoustic impedance of 2.23 MRayl at 200 °C. Metallic front face is an acoustic barrier to the transfer of acoustic energy in liquid sodium. Here, we show that a solution based on elastomeric materials is physically feasible and acoustically relevant.

### 5.2. Measurement of Velocities

In our test conditions, sound velocity in sodium at 200 °C was measured at 2407 m/s (2.6% lower than theoretical value of 2471 m/s).

Sound velocities in 316 L stainless steel at 200°C were determined at:

- V_L in 200°C SS_ = 5584 m/s
- V_S in 200°C SS_ = 3004 m/s

### 5.3. Further Work

#### 5.3.1. Time-Dependent Performances

The results shown in this article were reached during the first day of immersion of the transducer. It was noticed that performance slowly degraded day by day due to a loss of wetting of the front face. Nevertheless, the transducer can be repaired after reconditioning the elastomeric front face. Work is ongoing to understand the origin of this loss of wetting condition and to limit its effects.

#### 5.3.2. Under-Sodium Tests with Mockups Including Narrow Gap TIG Weld

Further work should be pursued in a similar study using a mockup, including a weld made by automated narrow gap TIG (Tungsten Inert Gas) welding. Indeed, such a weld will present a different geometrical and microstructural environment for the propagation of ultrasonic waves and thus disrupt the detection of artificial defects in a different way than the present X-shape SMAW weld.

#### 5.3.3. Irradiation

All the components and several material assemblies of the TUCSS transducers have been tested under irradiation up to 135 kGy without degradation. We can confidently assume that the whole transducer should be able to withstand 216 kGy (see specifications in Section 2), although a proper test under 200°C conditions is necessary to validate this point.

## 6. Conclusions

The experimental results obtained show that the new concept of TUCSS transducers exhibits sufficiently good acoustic properties to perform basic NDT of a structure immersed under liquid sodium at 200 °C, using conventional immersion ultrasonic technics.

The results obtained during this study demonstrate that conventional immersion ultrasonic NDT technics can be considered in this chemically aggressive environment during SFR outages. This work is an important step towards improved In-Service Inspection (ISI) of sodium-cooled nuclear reactors or other sodium related facilities.
